# Social capital and job satisfaction among substance abuse treatment employees

**DOI:** 10.1186/s13011-017-0093-6

**Published:** 2017-02-15

**Authors:** Andreas Tsounis, Dimitris Niakas, Pavlos Sarafis

**Affiliations:** 1 Centers for the Prevention of Addictions and Promoting Psychosocial Health of Municipality of Thessaloniki, Thessaloniki, Greece; 2 Hellenic Open University, Faculty of Social Sciences, Patra, Greece; 3 Department of Nursing, School of Health Sciences, Cyprus University of Technology, 15, Vragadinou Str, Limassol, 3041 Cyprus

**Keywords:** Social Capital, Job Satisfaction, Substance abuse, Employees

## Abstract

**Background:**

Job satisfaction is an important predictor for management and clinical ratios. Although it is accepted that is affected by many aspects, the influence of social capital remains to be determined. The main purpose of the article is to examine the relationship between job satisfaction and individual social capital for employees offering services in the treatment of addiction.

**Methods:**

A cross-sectional study was conducted on 239 employees from 14 therapeutic programs at KETEA (Therapy Center for Dependent Individuals in Greece) (KETHEA). A revised Greek Version of the Social Capital Questionnaire (SCQ-G) for the individual social capital measurement, and of the Job Satisfaction Survey (JSS) for the job satisfaction measurement, were used.

**Results:**

Individual Social Capital ranged in medium levels. We observed a significant positive association between social capital and its’ different aspects and gender, age, place of residence and working experience. Men, older employees, those who lived in smaller places, and those working more years, reached higher levels of individual social capital. Concerning overall job satisfaction most of the participants were ambivalent (61.5%), while 21.8% were satisfied and 16.7% were dissatisfied. Concerning its separate aspects, 77% were least satisfied with pay, 69.9% were least satisfied with advancement opportunities, 60.3% were least satisfied with fringe benefits, 85.8% were most satisfied with the nature of their work, 80.8% with their relationship with colleagues, and 77.8% were satisfied with supervision. Total Job Satisfaction was positively associated with place of residence and monthly salary. A significant positive correlation between social capital and job satisfaction was also observed.

**Conclusions:**

Early evidence suggests that social capital is associated with job satisfaction of employees providing services in the treatment of substance abuse. Further research, regarding social capital on job satisfaction, is suggested. We need to design and implement individual and organizational interventions for the empowerment of Social Capital.

## Background

Job satisfaction (JS) is defined as a positive personal perception of work or work related experiences [1]. It is a multidimensional experience influenced by several aspects, such as: salary, working conditions, workload, career development, interpersonal interactions, incentives, responsibilities and the nature of the work [2, 3]. It is one of the most frequently studied variables in organizational research [4], and is related to good organizational functioning [5]. Regarding the health service sector, JS is associated with higher standard of performance [6, 7], quality and efficiency of services and patient satisfaction [8]. It could also prevent occupational stress and turnover intention [3, 9], which is of great importance to the addiction treatment programs that suffer from turnover, with estimated annual rates of 18.5% [10].

Social capital (SC), on the other hand has been defined as “those features of social structures-such as interpersonal trust and norms of reciprocity and mutual aid–which act as resources for individuals and facilitate collective action” [11]. It can be regarded as a resource which helps people and organizations function, by lubricating interpersonal interactions and enabling cooperative ventures [11, 12].

There are two main theoretical and measurement approaches to SC: the individualistic and the communitarian. According to the first approach, people benefit from participating in social networks [13]. By participating, individuals gain access to material assets and to informational, emotional and appraisal support, that are key elements for personal development [13, 14]. In the second approach, SC features social systems that help members improve their health and their capacity to perform [12]. According to the collective approach, communities rich in social interactions, local networks and culture of participation in communal processes, present better economic, health and social indices [15].

Some additional distinctions are made between cognitive and structural, and bonding and bringing SC. Cognitive form refers to perceptions, beliefs and feelings like trust, reciprocity and tolerance. Structural SC explores how people behave in their social environment (e.g. level of participation, number of networks) [15]. Concerning the second distinction, bonding is defined as the SC within a group, which promotes reciprocity and homogeneity, while bringing occurs in SC between groups, which promotes information exchange and generates broader reciprocity and identification [11, 16].

Literature suggests that SC has a range of potential beneficial effects for individuals, local communities and society as a whole. A large body of research has found that its’ higher levels are related to better financial performance at both national and sub-national level [17, 18], lower levels of crime and social cohesion [19]. SC is also an important variable for educational attainment [20]. Inversely, lack of social capital is associated with negative health effects [21, 22]. In organizational studies, it has proved to be an important variable for both individual and organization success. It has contributed to career success, cross-functional team effectiveness, reducing turnover rates and facilitating entrepreneurship [23].

Concerning JS, literature suggests that it is significantly and positively affected by SC levels [12, 24, 25]. Basic SC components such as sense of trust, group solidarity, tolerance and sense of belonging improve workplace interactions. They help resolve work-related problems manage and motivate individuals to have faith and help each other. The above attitudes may affect the sense of JS, which is a key factor for both individual performance and organizational outcomes improvement.

Recourses that may boost employees’ performance and contribute to people’s wellbeing are of great interest, since the quality and the effectiveness of addiction treatment services depends a lot on this performance. SC is a resource which can help individuals and organizations to cope with work demands in many ways. It may either buffer the effects of burnout [26] or contribute to JS levels rising [12].

The aims of the present study are: (i) To measure individual SC among employees providing services in the treatment of substance abuse and to examine its’ relationship with a number of sociodemographic features. To our knowledge, there is no other relevant study. (ii) To measure JS levels in the specific population, and to examine its’ relationship with sociodemographic features. Although a large body of research has examined JS in various professions, only a few studies have assessed addiction treatment services employees’ JS [27, 28]. (iii) To estimate the associations of SC with JS, in an attempt of bridging a gap in the literature concerning the relation between SC and JS in the specific field. JS is a multidimensional occurrence, influenced by many variables. The study of parameters that have not been broadly studied is of great importance for developing organizational strategies that may enhance JS. This is crucial if we wish to have a higher standard of performance leading to an improvement in services provided.

## Methods

### Participants

The study was conducted in the Therapy Center for Dependent Individuals (KETHEA), which is the largest rehabilitation network for people facing substance use disorder and their families in Greece, from March through May 2015. KETHEA programs are drug-free and offer a comprehensive range of services including counseling and drug treatment, family support, health care, education and training, legal support and assistance reintegrating into society. The network of its’ services spreads in 23 cities and consists of 21 programs that include 100 units. Its’ personnel is composed of 520 employees [29]. Most staff members have a background in the social sciences, psychology and mental health and 20% are graduates of treatment programs [29]. Employees of all professional categories, including administrative, therapeutic and educational staff, part-time trainers and other specialties, compromised the sample. Questionnaires were distributed to a total of 341 employees. The final sample consisted of 239 professionals from 14 of the 21 programs.

### Procedure

The Research and Evaluation Committee of KETHEA reviewed and approved the research protocol and study questionnaire. Then permission was granted by all the scientific directors of all the programs. The scientific directors in turn asked all the employees if they wanted to participate in the study. 14 of the 21 programs responded positively. 6 from Athens (largest population in Greece), 3 from Thessaloniki (second largest) and 5 from other smaller cities. From the 7 programs that did not participate in the study, 5 answered that it was impossible due to schedule conflicts when the study was conducted and 2 declined without giving a specific reason. However, the sample could be considered a good representation, since bigger treatment programs were included, and because 341 out of 520 employees worked in the units that participated in the study. The questionnaires were posted and returned in sealed envelopes, one for every employee. The questionnaires were anonymous and filled out by each employee separately, while everyone received a brochure which explained the purpose of the research and encouraged them to participate in the study. The employees were informed that completing the questionnaire would be interpreted as informed consent. The whole procedure lasted 3 months.

### Measures

#### Socio-demographics

The first part of the questionnaire contained questions recording socio-demographic and work-related characteristics of the sample including sex, age, family status, place of residence, educational, specialty, professional experience and remuneration.

#### Social Capital Questionnaire-Greek version (SCQ-G)

Social Capital Questionnaire (SCQ) was developed in Australia by Onyx & Bullen. It has 36 questions in eight factors measuring different SC dimensions [30]. The SCQ has been validated in Greece (SCQ-G) comprising a total SC factor, as well as six factors in thirty-six questions: (i) Participation in the Local Community; (ii) Feelings of Safety; (iii) Family/Friends Connections; (iv) Value of Life and Social Agency; (v) Tolerance of Diversity; and (vi) Work Connections [31]. Each item ranked on a 4-point Likert scale. A single total score is derived from the scale as well as a score for each separate factor by adding the scores of the questions that best define each one of these factors. A higher score indicates more SC. Cronbach’s alpha was .85 for the whole SC scale and between 0.65 and 0.77 for the other factors, except Family/Friends Connections, that had an unacceptable low value of .29 and was not treated as a separate subscale in the subsequent analyses.

#### Job Satisfaction Survey (JSS)

Employees’ JS was assessed by Job Satisfaction Survey (JSS). JSS is a 36 item, with nine subscales, assessing employee attitudes about the job and its’ different aspects, which was developed by Paul Spector [32]. Each subscale is assessed with four items, while a total score is computed from all items. The subscales include (i) Pay; (ii) Promotion; (iii) Supervision; (iv) Fringe Benefits; (v) Contingent Rewards; (vi) Operating Procedures; (vii) Coworkers; (viii) Nature of Work, and (ix) Communication. JSS is a 6-point Likert-type scale and the respondents rate the favorable and unfavorable aspects of their jobs ranging from 1 (disagree very much) to (6 agree very much). Higher scores indicate higher JS. For the interpretation of the scores two approaches could be used: the normative and the absolute. Due to the fact that norms are not from representative samples and particularly for those countries that are not culturally similar with North America [33], the absolute approach was used in the current study. According to Spector instructions [33] for the 4-item subscales that range from 4 to 24, scores of 4 to 12 indicate dissatisfaction, 16 to 24 satisfaction and between 12 and 16 ambivalence. As far as the 36-item total scale, scores that range from 36 to 108 indicate dissatisfaction, from 144 to 216 satisfaction, and between 108 and 144 ambivalence [33]. Prior to the data collection, the English version of the JSS was translated into Greek. The forward–backward translation, which is the most commonly applied translation process for questionnaires or inventories [34], was performed. The final Greek version of the questionnaire was given to 12 volunteer participants for pilot testing. No translation errors, were detected. Cronbach alpha for the total scale was 0.87. Operating procedures was excluded in the present analysis because of its unacceptable value of .48. The reliability estimate for the other eight dimensions ranged from 0.62 to 0.87.

### Statistical analysis

The means and standard deviations were used to describe the levels of SC and JS of the participants. Independent sample t-test was used to compare total SC and total JS and their facets between two groups, in this case gender and one way ANOVA between three or more different groups, in this case all other socio-demographic features, were used. Pearson correlations coefficients were used to explore the association of total SC and total JS and their subscales. Correlation coefficient between 0.1 and 0.3 were considered low, between 0.31 and 0.5 moderate and those over 0.5 were considered high. Linear regression was further performed to estimate adjusted b-coefficients and 95% confidence intervals (95% CIs) respectively for the association between individual total SC and overall JS. All *P*-values were two- tailed, and *p* < 0.05 was considered to be statistically significant. Data analysis was performed using the statistical software SPSS version 20.0.

## Results

### Socio-demographic characteristics

A total of 239 participants were included in this analysis (70.09% response rate). The sample was predominantly female (64%), while the majority was aged between 40 and 50 years (45.2%) and was married (66.9%). Concerning educational level, 73.7% were university graduates. The majority worked as therapeutic staff (56.1%), followed by administrative staff (20.1%), education–research staff (12.1%), part-time trainers (7.5%) and other specialties (4.2%). Regarding professional experience, 37.2% of the participants worked between 11 and 15 years, 28% between 6 and 10 years, 13.8% between 16 and 20 years, 12.1% between 0 and 5 years, 7.1% between 20 and 25 years, while 1.7% worked for more than 26 years. The remuneration for the most employees (43.9%) ranged between 1000€ and 1300€ (Table 1).

**Table 1:** Demographic Features, Total SC and Total JS

			Total Social Capital			Total Job Satisfaction		
			High	Medium	Low	Satisfaction	Ambivalence	Dissatisfaction
	*N*	%	*N* (%)	*N* (%)	*N* (%)	*N* (%)	*N* (%)	*N* (%)
Gender								
Women	153	64	35 (22.9)	71 (46.4)	47 (30.7)	30 (19.6)	96 (62.7)	27 (17.6)
Men	86	36	30 (34.9)	42 (48.8)	14 (16.3)	22 (25.6)	51 (59.3)	13 (15.1)
Age (years)								
25–29	3	1.3	1 (33.3)	2 (66.7)	0 (0.0)	0 (0.0)	3 (100.0)	0 (0.0)
30–34	20	8.4	5 (25.0)	10 (50.0)	5 (25.0)	5 (25.0)	13 (65.0)	2 (10.0)
35–39	82	34.3	15 (18.3)	41 (50.0)	26 (31.7)	21 (25.6)	45 (54.9)	16 (19.5)
40–49	108	45.2	28 (25.9)	53 (49.1)	27 (25.0)	16 (14.8)	74 (68.5)	18 (16.7)
> 50	26	10.9	16 (61.5)	7 (26.9)	3 (11.5)	10 (38.5)	12 (46.2)	4 (15.4)
Family status								
Unmarried	66	27.6	21 (31.8)	29 (43.9)	16 (24.2)	14 (21.2)	44 (66.7)	8 (12.1)
Married	160	66.9	41 (25.6)	77 (48.1)	42 (26.2)	37 (23.1)	95 (59.4)	28 (17.5)
Divorced	9	3.8	2 (22.2)	5 (55.6)	2 (22.2)	1 (11.1)	6 (66.7)	2 (22.2)
Widowed	4	1.7	1 (25.0)	2 (50.0)	1 (25.0)	0 (0.0)	2 (50.0)	2 (50.0)
Place of Residence								
Athens	114	47.7	19 (16.7)	48 (42.1)	47 (41.2)	16 (14.0)	72 (63.2)	26 (22.8)
Thessaloniki	58	24.3	19 (32.8)	34 (58.6)	5 (8.6)	23 (39.7)	32 (55.2)	3 (5.2)
Other City	67	28	27 (40.3)	31 (46.3)	9 (13.4)	13 (19.4)	43 (64.2)	11 (16.4)
Educational level								
Post-graduate studies	91	38.1	22 (24.2)	44 (48.4)	25 (27.5)	17 (18.7)	53 (58.2)	21 (23.1)
University	85	35.6	19 (22.4)	46 (54.1)	20 (23.5)	21 (24.7)	57 (67.1)	7 (8.2)
Technological Institution	23	9.6	9 (39.1)	9 (39.1)	5 (21.7)	5 (21.7)	16 (69.6)	2 (8.7)
2 year Post Secondary	11	4.6	3 (27.3)	4 (36.4)	4 (36.4)	2 (18.2)	6 (54.5)	3 (27.3)
Upper Secondary	23	9.6	9 (39.1)	9 (39.1)	5 (21.7)	4 (17.4)	13 (56.5)	6 (21.6)
Low Secondary	6	2.5	3 (50.0)	1 (16.7)	2 (33.3)	3 (50.0)	2 (33.3)	1 (16.7)
Specialty								
Administrative staff	48	20.1	14 (29.2)	20 (41.7)	14 (29.2)	12 (25.0)	31 (64.6)	5 (10.4)
Therapeutic staff	134	56.1	36 (26.9)	67 (50.0)	31 (23.1)	26 (19.4)	84 (62.7)	24 (17.9)
Research staff	29	12.1	3 (10.3)	14 (48.3)	12 (41.4)	5 (17.2)	16 (55.2)	8 (27.6)
Part-time Trainers	18	7.5	8 (44.4)	10 (55.6)	0 (0.0)	6 (33.3)	11 (61.1)	1 (5.6)
Other Staff	10	4.2	4 (40.0)	2 (20.0)	4 (40.0)	3 (30.0)	5 (50.0)	2 (20.0)
Professional Experience								
0–5 years	29	12.1	8 (27.6)	15 (51.7)	6 (20.7)	9 (31.0)	16 (55.2)	4 (13.8)
6–10 years	67	28	13 (19.4)	37 (55.2)	17 (25.4)	13 (19.4)	43 (64.2)	11 (16.4)
11–15 years	89	37.2	20 (22.5)	40 (44.9)	29 (32.6)	18 (20.2)	57 (64.0)	14 (15.7)
16–20 years	33	13.8	10 (30.3)	17 (51.5)	6 (18.2)	5 (15.2)	20 (60.6)	8 (24.2)
20–25 years	17	7.1	10 (58.8)	4 (23.5)	3 (17.6)	4 (23.5)	10 (58.8)	3 (17.6)
> 26 years	4	1.7	4 (100.0)	0 (0.0)	0 (0.0)	3 (75.0)	1 (25.0)	0 (0.0)
Monthly Salary								
< 500€	18	7,5	8 (44.4)	10 (55.6)	0 (0.0)	6 (33.3)	11 (61.1)	1 (5.6)
501–800€	17	7,1	4 (23.5)	7 (41.2)	6 (35.3)	4 (23.5)	8 (47.1)	5 (29.4)
801–1000€	65	27,2	15 (23.1)	32 (49.2)	18 (27.7)	16 (24.6)	38 (58.5)	11 (16.9)
1001-1300€	105	43,9	28 (26.7)	49 (46.7)	28 (26.7)	15 (14.3)	70 (66.7)	20 (19.0)
1301–1600€	26	10,9	6 (23.1)	13 (50.0)	7 (26.9)	10 (38.5)	14 (53.8)	2 (7.7)
> 1601€	8	3,3	4 (50.0)	2 (25.0)	2 (25.0)	1 (12.5)	6 (75.0)	1 (12.5)

Individual Social capital has been classified as: the upper 27.2% as the high social capital group, the middle 47.3% as the medium and the lowest 25.5% as the low social capital group

### Social Capital

Mean values and standard deviations of employees’ individual SC and its subscales are provided in Table 2. Since there are no specific cut scores that determine whether an individual has low, medium or high SC, we cannot confidently put a dividing line between the different levels. However, by taking into account the range of the scores of the participants, we could say that means of total SC (mean = 90.79) and Participation in the Community (mean = 23.00) are in medium levels, while means of Value of Life and Social Agency (mean = 35.08), Feelings of Safety (mean = 5.82), Tolerance of Diversity (mean = 5.34) and Work Connections (mean = 11.44) range between medium and high.

**Table 2:** Means and standard deviations of employees individual SC and its subscales

	Social Capital		
	Mean	SD	Range
Participation in the Community	23.00	5.364	12–48
Value of Life and Social Agency	35.08	3.909	12–48
Feelings of Safety	5.82	1.151	2–8
Tolerance of Diversity	5.34	1.309	2–8
Work Connections	11.44	1.793	4–16
Total Social Capital	90.79	10.807	36–144

Socio-demographics, in relation to SC and its subscales are presented in Table 3. Concerning total SC, men (mean = 93.62), employees between 40 and 49 years (mean = 90.59), those who lived in smaller places (mean = 94.61) and those working more than 26 years (mean = 106.00), having higher scores. A t-test for gender and one-way ANOVA univariate analysis for all other demographics also revealed statistical significant associations between demographics and different aspects of SC (Table 3). Men expressed higher levels of Participation in the Community (mean = 24.51), Feelings of Safety (mean = 6.06) and improved Work Connections (mean = 11.90). Employees over 50 years old participated more in the community interactions (mean = 27.92). People living in smaller cities had higher scores concerning Participation in the community (mean = 24.48), Feelings of Safety (mean = 6.03) and Value of Life and Social Agency (mean = 36.54), while those living in Thessaloniki, which is a middle size city expressed higher scores as far as Work Connections evaluation (mean = 11.79). Most experienced employees (>26 years) participated more in local community interactions (mean = 32.50), but under this variable hides the older age that as we already show is associated with participation. Finally, educational level is positively associated with improved Work Connections, with those who had finished low (mean = 13.17) and upper (mean = 12.17) secondary education having higher scores. However, by taking into account that a large number from those employees who have completed only secondary education are treatment programs graduates we could say that the specific finding might be referred to them, but there is no clear evidence deriving from the statistical analysis. One way Anova did not reveal any significant difference in the cases of specialty and remuneration.

**Table 3:** Associations between SC and Its Subscales and Socio-demographic Characteristics

	Social Capital Scale (Greek-Version)											
	Participation in the Community		Value of Life and Social Agency		Feelings of Safety		Tolerance of Diversity		Work Connections		Total Social Capital	
	Mean	*p*	Mean	*p*	Mean	*p*	Mean	*p*	Mean	*p*	Mean	*p*
Gender		.002		.348		.017		.931		.002		.003
Women	22.16		34.90		5.69		5.33		11.18		89.21	
Men	24.51		35.38		6.06		5.35		11.90		93.62	
Age (years)		<.001		.229		.074		.646		.290		<.001
25–29	23.67		36.33		4.33		6.33		12.67		95.33	
30–34	21.40		35.55		5.65		5.40		11.60		89.85	
35–39	21.34		34.76		5.76		5.26		11.38		88.37	
40–49	23.36		34.82		5.85		5.32		11.29		90.59	
> 50	27.92		36.62		6.19		5.50		12.00		99.50	
Place of Residence		<.001		<.001		.029		.546		.012		<.001
Athens	21.42		33.68		5.61		5.25		11.08		86.90	
Thessaloniki	24.41		36.12		5.98		5.47		11.79		94.03	
Other (Smaller) City	24.48		36.54		6.03		5.39		11.75		94.61	
Educational level		.050		.578		.603		.247		.007		.115
Post-graduate studies	22.67		35.05		5.91		5.34		11.15		90.27	
University graduate	22.74		34.95		5.67		5.12		11.62		90.00	
Technological Institution	24.91		34.91		5.91		5.74		11.04		92.91	
2 year Post-Secondary	22.82		33.82		5.55		5.55		10.73		88.64	
Upper Secondary	22.00		35.83		5.96		5.70		12.17		91.74	
Low Secondary	28.67		37.17		6.17		5.17		13.17		102.17	
Professional Experience		<.001		.119		.562		.029		.772		<.001
0–5 years	22.14		35.17		5.66		5.62		11.34		90.52	
6–10 years	21.52		35.24		5.76		5.43		11.48		89.27	
11–15 years	22.43		34.39		5.78		4.98		11.29		88.73	
16–20 years	25.21		35.24		6.00		5.76		11.52		94.15	
20–25 years	26.82		36.76		6.24		5.53		12.00		98.00	
> 26 years	32.50		38.25		5.75		5.50		11.75		106.00	

Independent Samples T-test analysis was conducted for Gender and F-tests from one-way ANOVA was conducted for all other Socio-demographic features

*P* < 0.05

t and df for T-test:

Participation in the Community: 3.325, 237. Feelings of Safety: 2.422, 237. Work Connections: 2.997, 237. Total Social Capital: 3.079, 237

df for ANOVA:

Age: 4, 234. Place of Residence: 2, 236. Educational level: 5, 233 Professional Experience: 5, 233

### Job Satisfaction

Mean values and standard deviations of employees’ JS and its subscales are provided in Table 4. Means of overall JS (mean = 90.79), Contingent Rewards (mean = 13.99) and Communication (mean = 14.56) are in the area of ambivalence, scores for Pay (mean = 9.50), Promotion (mean = 10.11) and Fringe Benefits (mean = 11.58) reveal dissatisfaction and for Supervision (mean = 18.56), Coworkers (mean = 18.09) and Nature of Work (mean = 18.78) indicate satisfaction. The majority of the participants were least satisfied with the salaries (77%), the opportunities for advancement (69.9%) and the fringe benefits (60.3%), and most satisfied with the nature of work (85.8%) relationship with coworkers (80.8%) and supervision (77.8%).

**Table 4:** Means, standard deviations and rates (%) of employees total JS and its subscales

				Job Satisfaction		
	Mean	SD	Range	Satisfaction *N* (%)	Ambivalence *N* (%)	Dissatisfaction *N* (%)
Pay	9.50	3.61	4–24	12 (5)	43 (18)	184 (77)
Promotion	10.11	3.89	4–24	19 (7.9)	53 (22.2)	167 (69.9)
Supervision	18.56	4.59	4–24	186 (77.8)	26 (10.9)	27 (11.3)
Fringe Benefits	11.58	4.56	4–24	51 (21.3)	44 (18.4)	144 (60.3)
Contingent Rewards	13.99	4.11	4–24	93 (38.9)	63 (26.4)	83 (34.7)
Coworkers	18.09	3.32	4–24	193 (80.8)	33 (13.8)	13 (5.4)
Nature of Work	18.78	3.29	4–24	205 (85,8)	23 (9.6)	11 (4.6)
Communication	14.56	4.300	4–24	99 (41.4)	68 (28.5)	72 (30.1)
Total Job Satisfaction	128.26	20.54	36–216	52 (21.8)	147 (61.5)	40 (16.7)

Items Cut off Scores: 1–12: Dissatisfaction, 12–16: Ambivalence, 16–24: Satisfaction

Total Scale Cut off Scores: 36–108: Dissatisfaction, 108–144: Ambivalence, 144–216: Satisfaction

Socio-demographics, in relation to JS and its subscales are presented in Table 5. Residents of Thessaloniki (mean = 138.43) and employees with monthly salary between 1301 and 1600€ (mean = 136.38), where the most satisfied. One-way ANOVA univariate analysis also revealed statistical significant associations between demographics and different aspects of JS (Table 5). Employees living in Thessaloniki expressed higher levels of satisfaction as far as Promotion opportunities (mean = 10.98), Supervision (mean = 20.59), Contingent Rewards (mean = 15.55), relationships with Coworkers (mean = 19.07), Nature of Work (mean = 20.29) and Communication (mean = 16.48). Concerning educational level, employees who had completed secondary education, the majority of whom were graduates of treatment programs, expressed higher levels of satisfaction in the facets of Supervision (mean = 20.39 for Upper Secondary, mean = 20.00 for Low Secondary) and Nature of Work (mean = 19.70 for Upper Secondary, mean = 20.83 for Low Secondary), while those who had post-graduate studies were less satisfied in the case of Promotion policies (mean = 9.12) and Nature of Work (mean = 17.87). In the case of specialty, administrative staff expressed higher satisfaction levels as far as Pay (mean = 11.04) and Fringe Benefits (mean = 13.15), while part-time trainers were more satisfied with the Nature of Work (mean = 21.28) and their relationships with Coworkers (mean = 20.78). Professional experience was positively associated with Pay and Supervision, with employees working between 20 and 25 years being most satisfied in the first occasion (mean = 11.65) and those working more than 26 years in the second (mean = 20.75). Finally salary was significantly correlated with Pay, Supervision, Fringe Benefits, Coworkers, Nature of Work and Communication. Part time trainers who had monthly salary less than 500€ where most satisfied with all parameters except Pay and Fringe Benefits. T-test for Gender and One way Anova for age and family status did not reveal any significant difference.

**Table 5:** Associations between JS and Its Subscales and Socio-demographic Characteristics (One-way ANOVA)

	Job Satisfaction Survey (JSS)																	
	Pay		Promotion		Supervision		Fringe Benefits		Contingent Rewards		Coworkers		Nature of Work		Communication		Total Job Satisfaction	
	Mean	*p*	Mean	*p*	Mean	*p*	Mean	*p*	Mean	*p*	Mean	*p*	Mean	*p*	Mean	*P*	Mean	*p*
Place of Residence		.068		.039		<.001		.768		<.001		.017		<.001		<.001		<.001
Athens	8.99		9.47		17.89		11.54		12.99		18.01		17.93		13.85		123.32	
Thessaloniki	9.62		10.98		20.59		11.93		15.55		19.07		20.29		16.48		138.43	
Other (Smaller) City	10.27		10.45		17.94		11.34		14.34		17.39		18.91		14.09		127.88	
Educational level		.005		.042		.043		.826		.169		.384		.019		.974		.175
Post-graduate studies	9.53		9.12		17.57		11.31		13.45		17.76		17.87		14.29		124.22	
University	9.75		10.91		19.02		12.01		14.87		18.38		19.31		14.75		132.32	
Technological Institution	10.96		11.22		19.30		11.61		14.26		17.61		18.83		14.78		131.35	
2 year Post-Secondary	9.45		9.91		16.91		12.27		14.09		17.18		19.09		14.18		125.27	
Upper Secondary	6.87		10.00		20.39		11.09		12.87		19.13		19.70		14.91		127.30	
Low Secondary	10.17		10.50		20.00		10.17		12.83		18.67		20.83		14.33		129.50	
Specialty		<.001		.111		.091		.001		.998		.005		.001		.344		.815
Administrative staff	11.04		10.08		17.90		13.15		13.96		18.08		17.98		14.54		129.92	
Therapeutic staff	9.13		10.55		18.51		11.25		13.98		17.77		18.95		14.28		127.37	
Research staff	10.03		9.38		17.86		12.52		14.00		17.59		17.48		14.83		126.45	
Part-time Trainers	6.94		8.11		21.11		7.94		14.28		20.78		21.28		16.50		132.22	
Other Staff	10.20		10.10		19.80		12.30		13.80		19.10		19.60		14.00		130.50	
Professional Experience		.022		.647		.001		.145		.161		.119		.081		.063		.063
0–5 years	10.03		9.69		19.93		11.03		15.38		18.21		19.79		16.31		134.66	
6–10 years	8.61		9.79		19.31		10.60		14.22		18.64		18.61		14.79		127.93	
11–15 years	9.29		10.35		18.79		11.98		13.44		18.08		18.24		14.07		126.92	
16–20 years	10.06		9.91		16.06		11.76		13.42		17.48		19.18		13.64		124.18	
20–25 years	11.65		10.65		16.35		13.35		14.00		16.47		19.00		14.18		128.53	
> 26 years	11.50		12.75		20.75		14.25		17.00		20.25		22.00		18.00		150.00	
Monthly Salary		<.001		.133		<.001		<.001		.307		<.001		,004		.029		.037
< 500€	6.94		8.11		21.11		7.94		14.28		20.78		21.28		16.50		132.22	
501–800€	7.29		9.29		19.65		9.59		13.12		17.12		19.18		15.06		123.71	
801–1000€	9.40		10.12		20.00		11.45		14.18		18.95		18.92		15.14		131.85	
1001–1300€	9.65		10.23		17.54		11.86		13.72		17.49		18.12		13.59		124.58	
1301–1600€	11.88		11.35		18.00		13.92		15.46		18.04		19.46		15.62		136.38	
> 1601€	11.12		10.75		13.88		13.88		12.38		15.25		17.50		13.63		121.87	

F-tests from one-way ANOVA analysis was conducted

*p* < 0.05

df:

Place of Residence: 2, 236. Educational level: 5, 233 Specialty: 4, 234. Professional Experience: 5, 233. Monthly Salary: 5, 233

### Relationship between Social Capital and Job Satisfaction

In order to investigate the associations between SC and its subscales and overall JS and its subscales, a Pearson correlation was performed (Table 6). A significant positive association was observed between total SC and total JS (*p* = 0.002), indicating that higher individual SC is related with higher overall JS. However, correlation coefficient was considered low (*r* = 0.20). In addition, total SC score was positively significantly correlated with Promotion (*p* = 0.003, *r* = 0.191, *n* = 239), Contingent Rewards (*p* = 0.031, *r* = 0.139, *n* = 239), and Nature of Work (*p* = 0.000, r = 0.329, *n* = 239), while overall JS was positively significantly correlated with Value of Life and Social Agency (*p* = 0.007, *r* = 0.175, *n* = 239) and Work Connections (*p* = 0.000, *r* = 0.270, *n* = 239). Significant positive correlations were also observed between separate facets of each scale (Table 6).

**Table 6:** Correlation between SC and JS Scales

	Job Satisfaction (JSS)								
Social Capital (SCQ-G)	Pay	Promotion	Supervision	Fringe Benefits	Contingent Rewards	Coworkers	Nature of Work	Communication	Total Job Satisfaction
Participation in the Community	.159^*^	.210^*^	-.034	.087	.087	-.007	.219^*^	-.012	.123
Value of Life and Social Agency	.095	.062	.077	-.065	.173^*^	.098	.295^*^	.147^*^	.175^*^
Feelings of Safety	-.043	.058	.053	-.050	.065	.077	.143^*^	.145^*^	.108
Tolerance of Diversity	.075	.067	-.031	-.037	.017	.046	.100	-.004	.033
Work Connections	.007	.158^*^	.275^*^	-.023	.094	.296^*^	.403^*^	.218^*^	.270^*^
Total Social Capital	.124	.191^*^	.063	-.001	.139^*^	.101	.329^*^	.118	.200^*^

Pearson correlations analysis was conducted

^*^ *p* < 0.050

*n* = 239

**Fig. 1:**
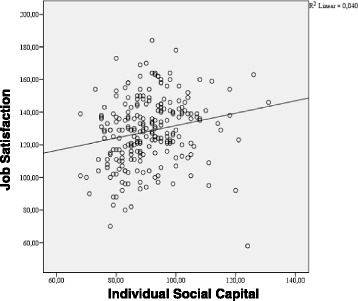
Association between Individual total SC and overall JS; Linear regression model

Linear regression model was further performed to estimate adjusted b-coefficients and 95% confidence intervals (95% CIs) respectively for the association between individual total SC and total JS. The model explained 4% of the JS variance (adjusted R^2^ = 0.040). According to the corresponding t-test, the standardized β was 0.20 and significant (*p* = 0.002, t = 3.145, df = 237) (Fig. 1).

## Discussion

The study investigated individual SC and JS levels among substance use disorder treatment employees and provided empirical support for the existence of their relationship.

Total SC and participation in the community facet were in medium levels, while value of life and social agency, feelings of safety, tolerance of diversity and work connections ranged between medium and high levels. Men, younger and older employees, those who lived in smaller cities and those who were most experienced, reported higher SC levels. Since the literature review did not reveal studies considering addiction treatment employees, the comparison with previous findings is not possible.

Concerning separate subscales, statistical analysis revealed a number of significant associations. Younger (<29) and older employees (>50) participated more in the life of local community compared with other age groups. At the same time, if we take into account that family status indirectly indicates age, we could say that younger employees are more tolerant in diversity, since unmarried (that are usually younger) reported higher levels of tolerance. Those living in smaller cities reported higher levels of participation in the local community, value of life and feelings of safety. Lower indices of criminality, stronger interpersonal relationships and more social interactions may interpret to some extent these findings. Finally, those working more than 26 years had higher total SC and more active involvement in local communities’ life, which is coherent with the above, since more than 25 years of professional experience indirectly indicate age, also.

Concerning overall JS the majority of the employees were ambivalent, while only 21.8% were satisfied. This is not consistent with previous findings in KETHEA in which workers expressed higher levels of satisfaction [35]. In addition, it doesn’t comply with the data of international literature, in which high levels of JS among workers in treatment programs were also expressed [27, 36–38]. Reductions in salaries, high turnover intention and raised therapeutic demands due to the financial crisis in Greece in the last 6 years, may justify the above findings [29].

As far as its different aspects, the majority of the participants were satisfied with the nature of work, which is consistent with past research data [27, 35, 38]. Satisfaction concerning relationship with coworkers and supervision were also in high levels. Supervision and administrative support have previously been shown to be related to JS, while management practices emphasizing support and fairness also benefit employee’s’ well-being [10, 39]. In addition, collegiate co-worker relationships and workplace social support are some of the most common sources of JS identified by drug treatment staff [38, 39].

Factors related with remuneration and lack of opportunities for career advancement have been identified as a significant source of dissatisfaction. The majority was dissatisfied with the salaries and the fringe benefits, which may be attributed to the income reduction that reached 30% during the past 5 years [29]. As far as career advancement, a number of past studies have identified the lack of opportunities for advancement as one of the most important sources of dissatisfaction for the employees in the specific field [27, 35, 40].

Statistical analysis also revealed a number of significant associations concerning the relation of socio-demographics and JS. Employees who had complete secondary education were more satisfied with supervision and nature of work, while those who had post-graduate studies were less satisfied with promotion policies and the nature of work. Of course in the case of addiction treatment programs job hierarchy and advancement is not directly connected with educational level, since part of the therapeutic staff is consisted from treatment programs graduates [29].

Employees’ specialty was positively correlated with pay and fringe benefits in the case of the administrative staff, and with coworkers and nature of work in the case of part-time trainers. This is in agreement with the findings of previous study in the same organization [35].

Length of service was also positively correlated with pay, supervision and promotion opportunities, with employees working more years, being more satisfied. This is in agreement with literature. In a national survey in which participated 1345 frontline Alcohol and Other Drug workers from treatment services across Australia, satisfaction levels were higher among those with more professional experience [38].

Finally part-time trainers, whom monthly salary is less than 500€, where most satisfied with supervision, coworkers, nature of work and communication issues, and less satisfied with remuneration (salary and fringe benefits) aspects. This can be interpreted from the fact that part-time trainers, the majority of whom are professors and other educators, are free from paperwork and other “bureaucratic issues” that have been identified by substance abuse workers as a significant source of dissatisfaction [35, 36].

According to the findings, there was a significant positive correlation between SC and JS. Nevertheless, literature review didn’t provide any study on addiction treatment field, while similar studies in other organizations are still limited.

However, our findings are consistent with the results of other studies on health-care organizations. In a study concerning the relationship among perceived organizational support, SC (interpersonal trust and institutional trust), health promotion and JS, in which 2884 employees of 16 hospitals in China participated, SC was found to be an important mediator between perceived organizational support and JS [41]. Another study, concerning the relationship between SC in hospitals and job satisfaction, in which 277 physicians from four German hospitals took part, showed that the SC of an organization, in addition to professional experience and workload, represents a significant predictor of physicians’ JS [11]. In a survey that took place among 959 nurses working at four hospitals in Germany, SC was negatively associated with emotional exhaustion [26]. Finally, the results of a study in Iran, showed that SC functioned as a factor of improvement of the level of communication, coordination and cooperation, that are significantly associated with JS [24].

As it was mentioned before, there are two forms of SC: individual and collective. In the above studies SC was measured as an ecological variable, reflecting the organizations’ function. So, the comparisons must be done under this prism, by taking into account this limitation.

Finally, similar data are reported in other working environments. The results of a study that was conducted among 315 Turkish school principals, confirmed that individual SC levels have positive and significant effect on JS [42]. Meanwhile, according to the findings of a study in which employees of a great variety of companies in Spain took part, higher levels of SC implied greater levels of satisfaction and quality of life at work. Specifically, SC was proved to be a better predictor of JS than the characteristics of the worker, the organization and the work environment [25].

JS is a key factor for both job performance and employee’s well-being [6], that may have a strong impact on the treatment results, which is the final outcome of a procedure that is taking place in a treatment program. SC is a multidimensional variable which reflects the value and the function of social networks and may affect many aspects that are related with JS levels. JS is depended on interpersonal relationships and SC may affect individuals’ attitudes. Consequently, future research could investigate the SC and JS relationship and inform our understanding of their association.

### Limitations

A number of limitations should be considered when interpreting the results of this study. First, the participants were selected on the basis of convenience, therefore, the extent to which the results could be generalized is limited. Second, due to unacceptable value of Cronbach alpha “Family/Friends Connections” factor, concerning SC and “Operating procedures” facet as far as JS, excluded from the analysis. Finally, data was collected during a period of great recession. Greece has lost 25% of the gross domestic product (GDP) [43]. This had a great impact on both the way organizations function and on the way individuals have adapted to the financial pressures, since under these conditions the professional attitudes and expectations are strongly affected.

## Conclusions

In summary, preliminary findings suggest that individual SC is positively associated with JS among drug addiction treatment employees. Further research, regarding both individuals’ and organizations’ SC on JS is suggested. Furthermore interventions for SC empowerment designed and implemented both on the individual and the organizational level (i.e. settings promoting interaction and cooperation among professionals, emphasizing trust, reciprocity and shared understanding) are required. Paying attention to SC theory may prove to be a useful approach for such interventions. A number of components of SC such as trust and mutual understanding may enable employees to act cooperatively and reinforce shared understanding and culture of collaboration, resulting in JS upgrading and occupational health improvement.

## Abbreviations

JS: Job satisfaction
KETHEA: Therapy Center for Dependent Individuals
SC: Social capital
